# Kahweol Inhibits Pro-Inflammatory Cytokines and Chemokines in Tumor Necrosis Factor-α/Interferon-γ-Stimulated Human Keratinocyte HaCaT Cells

**DOI:** 10.3390/cimb46040218

**Published:** 2024-04-18

**Authors:** Ye Jin Kwon, Hyun Hee Kwon, Jaechan Leem, Yoon Young Jang

**Affiliations:** 1 Department of Immunology, School of Medicine, Daegu Catholic University, Daegu 42472, Republic of Korea; kyjj0214@naver.com; 2 Department of Internal Medicine, School of Medicine, Daegu Catholic University, Daegu 42472, Republic of Korea; heeya0035@cu.ac.kr; 3 Department of Pediatrics, School of Medicine, Daegu Catholic University, Daegu 42472, Republic of Korea

**Keywords:** coffee, kahweol, atopic dermatitis, keratinocyte, cytokine, chemokine

## Abstract

Atopic dermatitis (AD), marked by intense itching and eczema-like lesions, is a globally increasing chronic skin inflammation. Kahweol, a diterpene that naturally occurs in coffee beans, boasts anti-inflammatory, antioxidative, and anti-cancer properties. This research explores the anti-inflammatory action of kahweol on HaCaT human keratinocytes stimulated by tumor necrosis factor-α (TNF-α) and interferon-γ (IFN-γ), focusing on key signal transduction pathways. Our results demonstrate that kahweol markedly reduces the production of IL-1β, IL-6, C-X-C motif chemokine ligand 8, and macrophage-derived chemokine in TNF-α/IFN-γ-activated HaCaT cells. Furthermore, it curtails the phosphorylation of key proteins in the mitogen-activated protein kinase (MAPK) pathways, including c-Jun N-terminal kinase, extracellular signal-regulated kinase, and p38. Additionally, kahweol impedes the phosphorylation and nuclear translocation of the NF-κB p65 subunit and constrains its DNA-binding capability. It also hampers the phosphorylation, nuclear translocation, and DNA-binding activities of signal transducer and activator of transcription 1 (STAT1) and STAT3. Collectively, these findings suggest that kahweol hinders the generation of cytokines and chemokines in inflamed keratinocytes by inhibiting the MAPK, NF-κB, and STAT cascades. These insights position kahweol as a promising agent for dermatological interventions, especially in managing inflammatory skin conditions such as AD.

## 1. Introduction

Atopic dermatitis (AD) is a long-lasting inflammatory skin disease, marked by severe itching and recurrent eczematous lesions [1,2]. Over the past three decades, AD’s prevalence has increased globally, affecting as much as 20% of individuals in developed nations [2]. AD often initiates the atopic march, leading to asthma and allergic rhinitis, and places a substantial psychosocial burden on patients and families [2]. Traditionally viewed as a simple skin condition, AD has been primarily treated with local anti-inflammatory treatments, including topical corticosteroids and calcineurin inhibitors [3]. For more severe cases, non-specific immunosuppressive drugs such as cyclosporine A, azathioprine, and methotrexate are used [4]. The recent introduction of treatments such as Janus kinase inhibitors, anti-IL-4Rα antibody, and anti-IL-13 antibody has expanded the options for moderate-to-severe AD [4,5]. Despite these advancements, the need for novel therapeutic agents remains critical.

The development of AD Involves intricate interactions between genetic, immunological, and environmental factors [6]. Its major pathological characteristics are skin barrier dysfunction and cutaneous inflammation [7]. Although it’s debated which factor initiates AD, it’s widely accepted that the compromise of the epidermal barrier and skin inflammation are interdependent and mutually reinforcing processes [8]. The epidermis, primarily consisting of keratinocytes, is crucial for maintaining skin barrier function, limiting water loss, and protecting against environmental allergens, chemicals, and microbial infections [9,10]. When the epidermal barrier is compromised, allergens and microbes can penetrate the skin, triggering inflammatory responses and activating keratinocytes. Pro-inflammatory cytokines further activate epidermal keratinocytes, causing them to produce more inflammatory mediators that draw immune cells like eosinophils and T cells to the area [11,12].

There has been significant interest in developing novel therapeutics from natural products known for their bioactive molecules with anti-inflammatory and antioxidative properties [13,14]. Natural biomolecules such as quercetin [15], resveratrol [16], and curcumin [17] have shown promise in treating inflammatory skin diseases like AD. Coffee ranks among the most popular beverages globally, and there’s growing evidence linking its consumption to a reduced risk of several health disorders [18]. These include neurodegenerative diseases, chronic liver disease, type 2 diabetes, cancers, and cardiovascular diseases [18,19,20,21]. Notably, recent research has found a negative association between coffee consumption and the prevalence of AD in Korean adults [22]. Additionally, Barrea et al. reported that there was a negative correlation between the clinical severity of psoriasis, another inflammatory skin disease, and coffee consumption [23]. Coffee is known to contain a multitude of bioactive substances, such as caffeine, various polyphenols, and diterpenes, contributing to its unique properties [18]. One of the primary diterpenes in coffee beans is kahweol, which possesses diverse biological properties, including anti-inflammatory, antioxidative, and anticancer effects [24,25]. Studies demonstrate kahweol’s therapeutic efficacy in various inflammation models, including carrageenan-induced paw edema [26], carbon tetrachloride-induced hepatotoxicity [27], thioacetamide-induced liver fibrosis [28], and traumatic brain injury [29]. Our recent work has also shown kahweol’s effectiveness in alleviating cisplatin-induced nephrotoxicity [30] and acetaminophen-induced hepatotoxicity [31] in mice. However, the impact of kahweol on skin inflammation remains unexplored.

This study specifically aims to investigate the impact of kahweol on cytokine and chemokine production in human keratinocyte HaCaT cells inflamed by tumor necrosis factor-α (TNF-α) and interferon-γ (IFN-γ). By delving into the mechanisms behind these effects, we hope to elucidate how kahweol might modulate skin inflammation at a cellular level, potentially offering a new therapeutic avenue for AD management. This research seeks to bridge the gap between the known systemic benefits of kahweol and its unexplored potential in dermatological applications.

## 2. Materials and Methods

### 2.1. Cell Culture

The HaCaT human keratinocyte cell line was grown in high-glucose DMEM, supplemented with 10% fetal bovine serum. These cells were maintained at 37 °C in a humidified incubator with 5% CO_2_. For experimental procedures, the HaCaT cells were seeded in 60 mm dishes at a density of 5.0 × 10^5^ cells per dish and maintained in a complete medium. After 24 h, the medium was substituted with serum-free DMEM, to which kahweol was added at concentrations of 5 or 10 μM. The kahweol was solubilized in DMSO, ensuring that the concentration of the solvent in the final treatment medium did not exceed 0.5% (*v*/*v*). Subsequent to a one-hour incubation period with kahweol, the cells were treated with TNF-α (10 ng/mL; R&D Systems, Minneapolis, MN, USA) and IFN-γ (10 ng/mL; R&D Systems). This stimulation was carried out for specified periods at 37 °C, after which both cells and supernatants were collected for subsequent analysis.

### 2.2. Cell Viability Assay

The viability of HaCaT cells was assessed using the Cell Counting Kit (CCK)-8 assay (Dojindo, Kumamoto, Japan), following the manufacturer’s protocols. Briefly, the cells were plated into 96-well plates at a density of 5.0 × 10^3^ cells per well and allowed to stabilize for 24 h. Subsequently, the cells were exposed to various concentrations of kahweol (1, 5, 10, 25, and 50 μM) for 24 h. Then, 10 mL of a water-soluble tetrazolium salt (WST-8) solution was added to each well. An additional incubation of 4 h at 37 °C was performed. The cell viability was subsequently assessed by measuring the absorbance at 450 nm with a microplate reader (Thermo Fisher Scientific, Waltham, MA, USA).

### 2.3. Quantitative Real-Time Polymerase Chain Reaction (qPCR)

Total RNA was isolated from the cultured cells utilizing TRIzol reagent (Thermo Fisher Scientific). The conversion of RNA into cDNA was carried out with the RNA to cDNA EcoDry^TM^ Premix Kit (TaKaRa, Tokyo, Japan), following the manufacturer’s instructions. The qPCR analysis was executed using the Thermal Cycler Dice Real Time System III (TaKaRa) and the Power SYBR Green PCR Master Mix (TaKaRa), following the manufacturer’s protocols. The primers used in this research are detailed in Table 1. Relative mRNA transcription levels were quantified using the 2^−ΔΔCt^ method, with glyceraldehyde-3-phosphate dehydrogenase (GAPDH) being used as the internal control for normalization.

### 2.4. Enzyme-Linked Immunosorbent Assay (ELISA)

The concentration levels of IL-1β, IL-6, C-X-C motif chemokine ligand 8 (CXCL8), and macrophage-derived chemokine (MDC) in the cell culture supernatants were analyzed using ELISA kits (R&D Systems), following the manufacturer’s protocols. The optical densities of these assays were ascertained at 450 nm using a microplate reader (Thermo Fisher Scientific).

### 2.5. Western Blot Analysis

The cells were lysed by using a lysis buffer for 30 min on ice. The lysates were subjected to centrifugation at 13,000 rpm for 10 min at 4 °C, and the resulting supernatants were collected. The protein concentrations in these supernatants were determined using a BCA assay kit (Thermo Fisher Scientific). The proteins were then loaded onto gradient polyacrylamide gels for separation and subsequently transferred onto nitrocellulose membranes. These membranes were blocked with 5% skim milk in Tris-buffered saline with Tween 20 (TBS-T) at room temperature for 1 h. They were then incubated with primary antibodies targeting c-Jun N-terminal kinase (JNK), p-JNK, extracellular signal-regulated kinase (ERK), p-ERK, p38, p-p38, NF-κB p65, p-NF-κB p65, signal transducer and activator of transcription 1 (STAT1), p-STAT1, STAT3, p-STAT3, and GAPDH, all sourced from Cell Signaling Technology (Danvers, MA, USA). Following three 10 min washes in TBS-T on a shaker, the membranes were exposed to horseradish peroxidase-conjugated secondary antibodies for 1 h at room temperature. Following repeated washing steps, the detection was performed using enhanced chemiluminescence reagents and visualized under the iBright CL1500 Imaging System (Thermo Fisher Scientific). The band densities were quantified using ImageJ software Version 1.53j, and the protein expression levels were normalized against GAPDH.

### 2.6. Electrophoretic Mobility Shift Assay (EMSA)

The EMSA was performed as described previously [32]. Briefly, the process began with the extraction of the nuclear fraction from cells using the NE-PER Nuclear and Cytoplasmic Extraction Kit (Thermo Fisher Scientific). The EMSA was then carried out using the Lightshift^TM^ Chemiluminescent EMSA Kit (Thermo Fisher Scientific), following the manufacturer’s protocols. In this study, specific oligonucleotide probes were used: for NF-κB, the sequence was 5′-AGT TGA GGG GAC TTT CCC AGG C-3′; for STAT1, it was 5′-CAT GTT ATG CAT ATT CCT GTA AGT G-3′; and for STAT3, the sequence used was 5′-GAT CCT TCT GGG AAT TCC TAG ATC-3′. The detection of the biotin-labeled DNA’s chemiluminescent signal was performed using the iBright CL1500 Imaging System (Thermo Fisher Scientific).

### 2.7. Immunofluorescence (IF) Staining

The cells underwent fixation with 4% paraformaldehyde for a duration of 10 min at room temperature. Post-fixation, they were treated with 0.1% Triton X-100 in PBS for permeabilization, also lasting 10 min. The permeabilized cells were then blocked for 1 h at room temperature using PBS mixed with 5% bovine serum albumin. The subsequent step involved an overnight incubation at 4 °C with primary antibodies directed against NF-κB, STAT1, or STAT3. Following this, the cells were incubated with FITC or Alexa FluorTM 488-conjugated secondary antibodies (Invitrogen, Carlsbad, CA, USA) for 2 h at room temperature. Afterwards, the cell nuclei were stained with DAPI. For microscopic analysis, the slides were prepared using a fluorescence mounting medium, and images were captured using a confocal microscope (Nikon, Tokyo, Japan).

### 2.8. Statistical Analysis

The data were presented as the mean ± SEM. Group differences were analyzed using one-way ANOVA, followed by Tukey’s multiple comparison test. Statistical significance was determined based on a *p*-value of less than 0.05.

## 3. Results

### 3.1. Impact of Kahweol on the Viability of Human Keratinocyte HaCaT Cells

**Figure 1:**
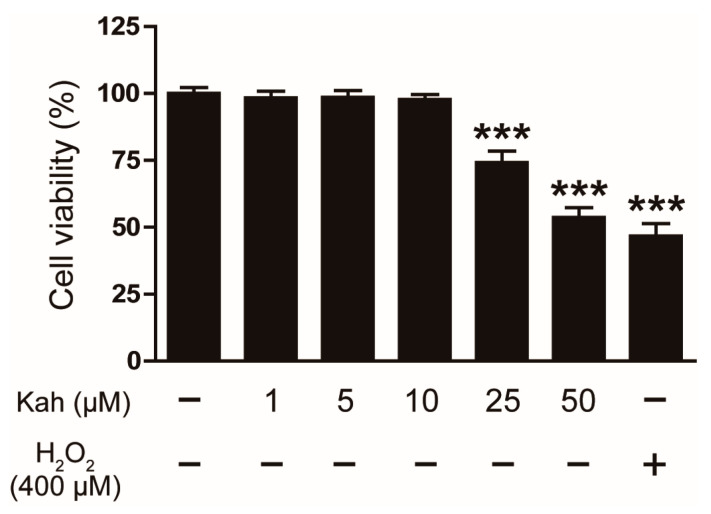
The cytotoxicity of kahweol in human keratinocytes. The HaCaT cells were exposed to kahweol (1, 5, 10, 25, and 50 μM) for 24 h. The cell viability was evaluated using the CCK-8 assay. H_2_O_2_ (400 μM) was used as a positive control. The data are presented as mean ± SEM of three independent experiments. *** *p* < 0.001 versus the untreated control group.

We first examined the cytotoxic effects of kahweol on the HaCaT cells using the CCK-8 assay. Various concentrations of kahweol (1, 5, 10, 25, and 50 μM) were administered to the cells for 24 h. It was noted that kahweol did not significantly impact the viability of the HaCaT cells at concentrations up to 10 μM (Figure 1). Therefore, subsequent experiments used kahweol concentrations of 5 and 10 μM, which were deemed non-toxic to the cells.

### 3.2. Impact of Kahweol on Cytokine and Chemokine Production in TNF-α/IFN-γ-Activated Keratinocytes

**Figure 2:**
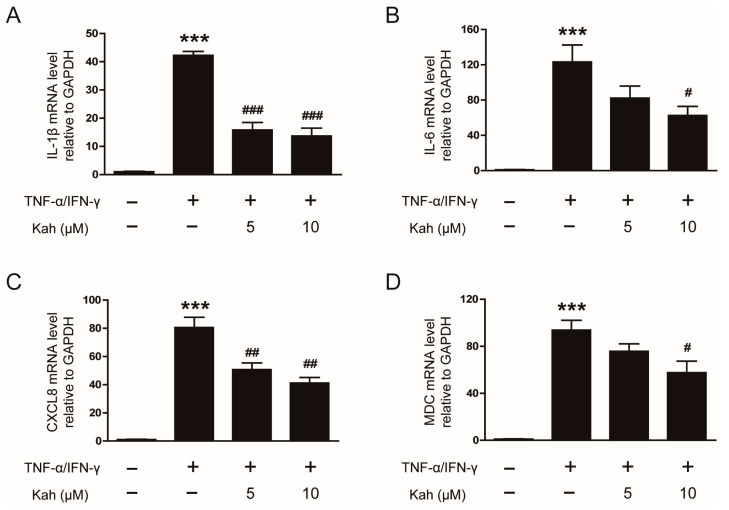
The impact of kahweol on mRNA levels of cytokines and chemokines in TNF-α/IFN-γ-activated human keratinocytes. The HaCaT cells underwent a pre-treatment with kahweol at concentrations of 5 and 10 μM for 1 h, followed by stimulation using TNF-α/IFN-γ (10 ng/mL each) for 24 h. The relative mRNA levels of (**A**) IL-1β, (**B**) IL-6, (**C**) CXCL8, and (**D**) MDC were analyzed using qPCR. The data are presented as mean ± SEM of three independent experiments. *** *p* < 0.001 versus the group treated with vehicle (DMSO) alone. ^#^ *p* < 0.05, ^##^ *p* < 0.01, and ^###^ *p* < 0.001 versus the group treated with TNF-α/IFN-γ alone.

To evaluate the anti-inflammatory activity of kahweol, we examined cytokine (IL-1β and IL-6) and chemokine (CXCL8 and MDC) production in the TNF-α/IFN-γ-stimulated HaCaT cells. Treatment with TNF-α and IFN-γ (10 ng/mL each) markedly elevated the mRNA expression of IL-1β, IL-6, CXCL8, and MDC in keratinocytes (*p* < 0.001 for all; Figure 2A–D). However, pretreatment with kahweol at 5 and 10 μM concentrations notably reduced the mRNA levels of these inflammatory markers. Kahweol significantly decreased IL-1β mRNA levels by 62.7% at 5 μM and 67.6% at 10 μM (*p* < 0.001 for both; Figure 2A), and reduced IL-6 by 49.2% at 10 μM (*p* < 0.05; Figure 2B). It also decreased CXCL8 mRNA levels by 37.0% at 5 μM and 48.8% at 10 μM (*p* < 0.01 for both; Figure 2C), and reduced MDC by 38.6% at 10 μM (*p* < 0.05; Figure 2D).

**Figure 3:**
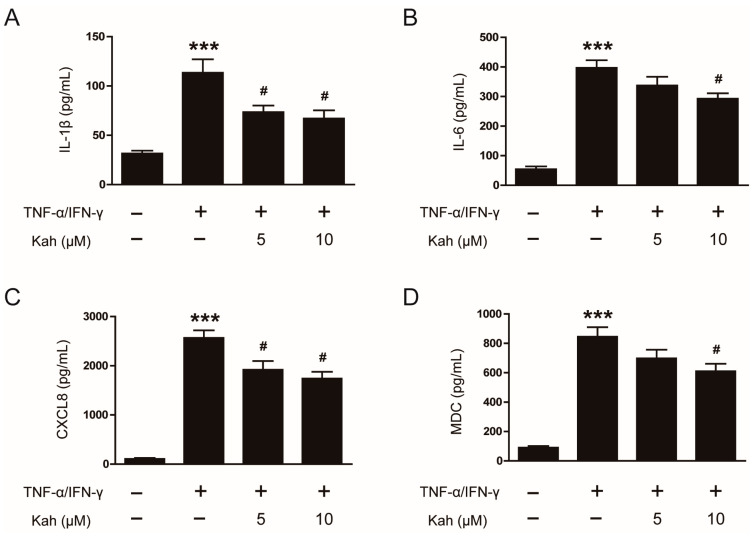
The impact of kahweol on the production of cytokines and chemokines in TNF-α/IFN-γ-activated human keratinocytes. The HaCaT cells underwent a pre-treatment with kahweol at concentrations of 5 and 10 μM for 1 h, followed by stimulation using TNF-α/IFN-γ (10 ng/mL each) for 24 h. The protein levels of (**A**) IL-1β, (**B**) IL-6, (**C**) CXCL8, and (**D**) MDC were measured in culture supernatants using the ELISA analysis. The data are presented as mean ± SEM of three independent experiments. *** *p* < 0.001 versus the group treated with vehicle alone. ^#^ *p* < 0.05 versus the group treated with TNF-α/IFN-γ alone.

Further analysis using ELISA on the culture supernatants showed that treatment with TNF-α and IFN-γ elevated the protein levels of IL-1β, IL-6, CXCL8, and MDC (*p* < 0.001 for all; Figure 3A–D). However, pretreatment with kahweol at concentrations of 5 and 10 μM led to a notable decrease in the levels of these proteins. Specifically, IL-1β levels were lowered by 35.2% at 5 μM and 40.9% at 10 μM (*p* < 0.05 for both; Figure 3A). Similarly, the levels of IL-6 decreased by 26.4% at 10 μM (*p* < 0.05; Figure 3B). Reductions were also observed in CXCL8, with a decrease of 25.1% at 5 μM and 32.2% at 10 μM (*p* < 0.05 for both; Figure 3C), and MDC, which diminished by 27.8% at 10 μM (*p* < 0.05; Figure 3D). These results suggest that kahweol exhibits anti-inflammatory activity on inflamed keratinocytes, as evidenced by its ability to reduce the generation of key inflammatory mediators.

### 3.3. Impact of Kahweol on Mitogen-Activated Protein Kinase (MAPK) Signaling Cascades in TNF-α/IFN-γ-Activated Keratinocytes

**Figure 4:**
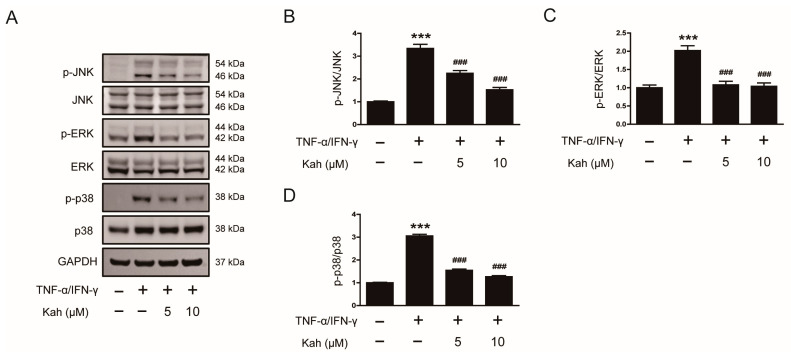
The impact of kahweol on MAPK signaling cascades in TNF-α/IFN-γ-activated human keratinocytes. The HaCaT cells underwent a pre-treatment with kahweol at concentrations of 5 and 10 μM for 1 h, followed by stimulation using TNF-α/IFN-γ (10 ng/mL each) for 5 min. (**A**) Western blotting of p-JNK, p-ERK, and p-p38. The bar graphs represent quantitative band densities of (**B**) p-JNK, (**C**) p-ERK, and (**D**) p-p38. The data are presented as mean ± SEM of three independent experiments. *** *p* < 0.001 versus the group treated with vehicle alone. ^###^ *p* < 0.001 versus the group treated with TNF-α/IFN-γ alone.

The MAPK cascades, which include JNK, ERK, and p38 pathways, play a crucial role in modulating inflammatory responses in keratinocytes [33,34]. The protein levels of JNK, ERK, and p38 in their phosphorylated forms showed a significant increase 5 min following the treatment of TNF-α and IFN-γ (*p* < 0.001 for all; Figure 4A–D). However, pretreatment with kahweol at 5 and 10 μM concentrations resulted in a reduction in the phosphorylation of these proteins. Specifically, kahweol decreased JNK phosphorylation by 32.6% at 5 μM and 54.2% at 10 μM (*p* < 0.001 for both; Figure 4A,B), ERK phosphorylation by 46.5% at 5 μM and 48.5% at 10 μM (*p* < 0.001 for both; Figure 4A,C), and p38 phosphorylation by 49.3% at 5 μM and 58.5% at 10 μM (*p* < 0.001 for both; Figure 4A,D). These results demonstrate that kahweol effectively inhibits the MAPK signaling cascades in keratinocyte under inflammatory conditions.

### 3.4. Impact of Kahweol on NF-κB Activation in TNF-α/IFN-γ-Activated Keratinocytes

**Figure 5:**
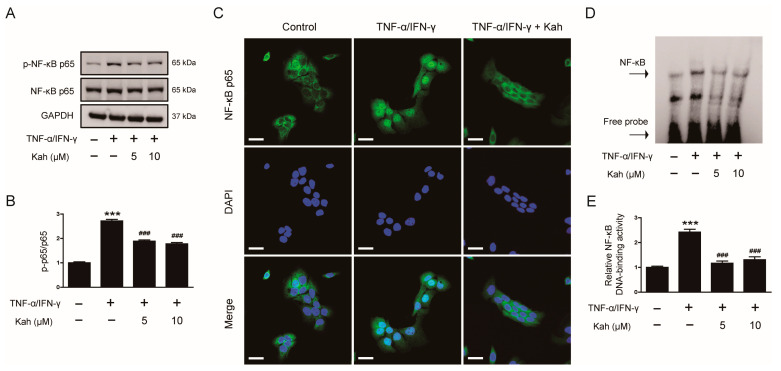
The impact of kahweol on the TNF-α/IFN-γ-evoked NF-κB activation in human keratinocytes. (**A**) Western blotting of p-NF-κB p65. The HaCaT cells underwent a pre-treatment with kahweol at concentrations of 5 and 10 μM for 1 h, followed by stimulation using TNF-α/IFN-γ (10 ng/mL each) for 15 min. (**B**) The bar graph represents quantitative band densities of p-NF-κB p65. (**C**) IF staining for NF-κB p65. HaCaT cells underwent a pre-treatment with 10 μM kahweol for 1 h, followed by stimulation using TNF-α/IFN-γ (10 ng/mL each) for 30 min. Nuclei were visualized using DAPI. Scale bars: 25 μm. (**D**) EMSA for NF-κB DNA-binding activity. HaCaT cells underwent a pre-treatment with kahweol at concentrations of 5 and 10 μM for 1 h, followed by stimulation using TNF-α/IFN-γ (10 ng/mL each) for 30 min. (**E**) The bands corresponding NF-κB were quantified by densitometry. The data are presented as mean ± SEM of three independent experiments. *** *p* < 0.001 versus the group treated with vehicle alone. ^###^ *p* < 0.001 versus the group treated with TNF-α/IFN-γ alone.

NF-κB is a transcription factor crucial for modulating the expression of inflammatory mediators [35,36]. In TNF-α/IFN-γ-activated keratinocytes, NF-κB p65 phosphorylation significantly increased at 15 min (*p* < 0.001; Figure 5A,B). Kahweol pretreatment reduced NF-κB p65 phosphorylation by 30.5% at 5 μM and 34.6% at 10 μM (*p* < 0.001 for both; Figure 5A,B). IF staining showed that pretreatment with 10 μM kahweol effectively hindered the nuclear transport of NF-κB p65 (Figure 5C). Furthermore, EMSA results indicated that kahweol decreased NF-κB DNA-binding activity by 51.7% at 5 μM and 45.9% at 10 μM in the stimulated cells (*p* < 0.001 for both; Figure 5D,E). These findings suggest that kahweol is effective in inhibiting the activation of NF-κB in inflamed keratinocytes.

### 3.5. Impact of Kahweol on STAT1 and STAT3 Activation in TNF-α/IFN-γ-Activated Keratinocytes

**Figure 6:**
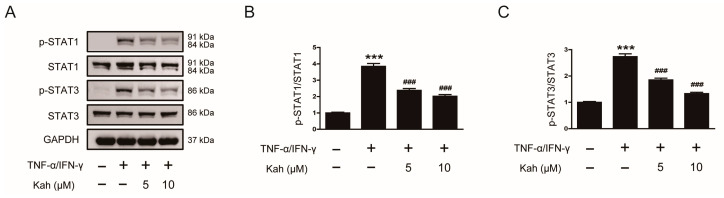
The impact of kahweol on the TNF-α/IFN-γ-evoked phosphorylation of STAT1 and STAT3 in human keratinocytes. (**A**) Western blotting of p-STAT1 and p-STAT3. The HaCaT cells underwent a pre-treatment with kahweol at concentrations of 5 and 10 μM for 1 h, followed by stimulation using TNF-α/IFN-γ (10 ng/mL each) for 30 min. The bar graphs represent quantitative band densities of (**B**) p-STAT1 and (**C**) p-STAT3. The data are presented as mean ± SEM of three independent experiments. *** *p* < 0.001 versus the group treated with vehicle alone. ^###^ *p* < 0.001 versus the group treated with TNF-α/IFN-γ alone.

STAT proteins are a group of transcription factors critical for mediating cytokine signal transduction [37]. The activation of STAT1 and STAT3 within keratinocytes plays a significant role in the development of skin inflammation [38]. When HaCaT cells were subjected to TNF-α and IFN-γ, there was a marked increase in the phosphorylation of both STAT1 and STAT3 (*p* < 0.001 for each; Figure 6A–C). The pretreatment of these cells with kahweol reduced STAT1 phosphorylation by 38.2% at 5 μM and 47.5% at 10 μM (*p* < 0.001 for both; Figure 6A,B). Additionally, kahweol was effective in inhibiting STAT3 phosphorylation by 49.7% at 5 μM and 51.5% at 10 μM (*p* < 0.001 for both; Figure 6A,C).

**Figure 7:**
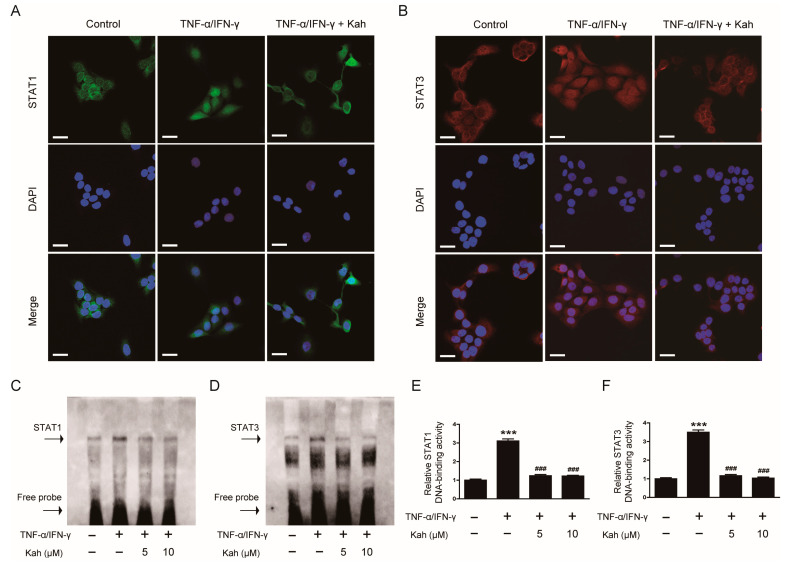
The impact of kahweol on the nuclear translocation and DNA-binding activities of STAT1 and STAT3 in TNF-α/IFN-γ-activated human keratinocytes. IF staining for (**A**) STAT1 and (**B**) STAT3. The HaCaT cells underwent a pre-treatment with 10 μM kahweol for 1 h, followed by stimulation using TNF-α/IFN-γ (10 ng/mL each) for 2 h. Nuclei were visualized using DAPI. Scale bars: 25 μm. EMSA for the DNA-binding activities of (**C**) STAT1 and (**D**) STAT3. The HaCaT cells underwent a pre-treatment with kahweol at concentrations of 5 and 10 μM for 1 h, followed by stimulation using TNF-α/IFN-γ (10 ng/mL each) for 30 min. The bands corresponding (**E**) STAT1 and (**F**) STAT3 were quantified by densitometry. The data are presented as mean ± SEM of three independent experiments. *** *p* < 0.001 versus the group treated with vehicle alone. ^###^ *p* < 0.001 versus the group treated with TNF-α/IFN-γ alone.

IF staining demonstrated that 10 μM of kahweol effectively suppressed the nuclear translocation of STAT1 induced by TNF-α/IFN-γ (Figure 7A). Additionally, the increased nuclear transport of STAT3 observed in the TNF-α/IFN-γ-activated HaCaT cells was similarly suppressed by 10 μM of kahweol (Figure 7B). EMSA results indicated that treating keratinocytes with TNF-α and IFN-γ enhanced DNA-binding activities of STAT1 (Figure 7C,E) and STAT3 (Figure 7D,F). However, pretreatment with kahweol effectively reduced the DNA-binding activity of STAT1 by 60.1% at 5 μM and 60.8% at 10 μM (*p* < 0.001 for both; Figure 7C,E). Similarly, kahweol inhibited the DNA-binding activity of STAT3 by 66.6% at 5 μM and 70.3% at 10 μM (*p* < 0.001 for both; Figure 7D,F). These findings indicate that kahweol effectively suppresses the activation of STAT1 and STAT3 in inflamed keratinocytes.

## 4. Discussion

This study demonstrated that kahweol treatment decreased the generation of cytokines and chemokines in TNF-α/IFN-γ-activated human keratinocytes. This effect was achieved by inhibiting the MAPK, NF-κB, and STAT signaling cascades.

The anti-inflammatory properties of kahweol on skin inflammation were examined in this study using a HaCaT cell model treated with TNF-α and IFN-γ. This approach was chosen based on several critical aspects of AD and the role of keratinocytes in this disease. Keratinocytes, known to be central in AD pathogenesis, are activated by various cytokines from different immune cells [11]. Among these, TNF-α and IFN-γ are the most recognized for their role in keratinocyte activation [10]. Once activated, keratinocytes produce numerous cytokines, including IL-1β and IL-6, which intensify the inflammatory response through autocrine and paracrine actions [10,39]. Moreover, when HaCaT cells are co-stimulated with TNF-α and IFN-γ, it triggers the generation of Th-2-related chemokines such as CXCL8 and MDC [40,41]. These chemokines are crucial in recruiting inflammatory cells to skin lesions, thus contributing to the progression from acute to chronic inflammatory responses [42]. Therefore, the TNF-α/IFN-γ-stimulated HaCaT cell model serves as a pertinent and efficient system to study the potential therapeutic impact of kahweol on inflammatory skin diseases, especially AD. The findings of this study showed that pretreatment with kahweol markedly reduces the generation of cytokines (IL-1β and IL-6) and chemokines (CXCL8 and MDC) in TNF-α/IFN-γ-activated keratinocytes.

TNF-α and IFN-γ activate various intracellular signaling cascades, including the MAPK pathways, in keratinocytes [34]. The MAPK family comprises three major groups: JNK, ERK, and p38 [43,44]. Upon exposure to TNF-α and IFN-γ, the MAPK pathways in keratinocytes become activated. This activation results in the phosphorylation of JNK, ERK, and p38, leading to the production of pro-inflammatory mediators [34]. Our study found that kahweol effectively reduced the phosphorylation of JNK, ERK, and p38 in keratinocytes stimulated with TNF-α/IFN-γ, suggesting its inhibitory effects on the MAPK signaling pathways. This is consistent with prior studies, which have shown that decreasing the phosphorylation of MAPK molecules can reduce inflammatory responses in keratinocytes [45,46,47]. Besides influencing cytokine and chemokine release, MAPK pathways participate in several cellular functions, including cell proliferation, differentiation, apoptosis, and survival [43]. The JNK pathway in particular is essential in regulating critical functions such as keratinocyte proliferation, differentiation, and apoptosis [48]. Its activation in inflammatory skin conditions disrupts the skin barrier, leading to dryness and irritation. On the other hand, the ERK pathway predominantly manages keratinocyte proliferation and survival [49,50]. In AD, dysregulated ERK signaling is linked to abnormal keratinocyte proliferation and compromised skin barrier function, both key factors in the development of chronic AD lesions [51]. Meanwhile, the p38 MAPK pathway is closely associated with keratinocyte differentiation and apoptosis, significantly contributing to the pathophysiology of skin inflammation [52,53].

NF-κB, a key molecule downstream of the MAPK pathway, plays a crucial role in the production of cytokines and chemokines [54]. The MAPK pathways trigger the transport of NF-κB from the cytosol to the nucleus via a series of phosphorylation events [43]. This translocation is typically facilitated by the degradation or modification of IκB, which normally keeps NF-κB inactive in the cytosol [54]. Once inside the nucleus, NF-κB attaches to specific DNA sequences, initiating the transcription of various genes, including those coding for cytokines, chemokines, adhesion molecules, and enzymes that modulate inflammatory responses [35,36]. In this study, it was noted that kahweol effectively suppressed NF-κB activation in keratinocytes activated with TNF-α/IFN-γ. This effect was marked by a notable decrease in both the phosphorylation and nuclear transport of NF-κB p65, as well as a reduction in NF-κB’s DNA-binding activity. Aligning with our results, prior studies indicate that the compound attenuates lipopolysaccharide-triggered NF-κB activation and inflammatory gene transcription in macrophages [55,56]. Additionally, kahweol has been found to inhibit TNF-α-evoked upregulation of adhesion molecules in endothelial cells by hindering NF-κB activation [57]. Furthermore, it suppresses the production of cytokines and the activation of NF-κB in primary mouse Kupffer cells and hepatocytes [58]. Notably, kahweol also enhances pancreatic β-cell function by inhibiting the NF-κB cascade [59].

STAT proteins are essential for transmitting extracellular cytokine signals to the cell nucleus, thus influencing gene expression [37]. They play a crucial role in the development of AD and its associated inflammation [38]. STAT1 activation aggravates AD by upregulating genes linked to inflammation, often triggered by IFNs and other cytokines [38]. On the other hand, STAT3 has a dual role: it contributes to AD’s inflammation and itching but is also vital for skin barrier integrity and wound healing [60,61,62]. Thus, the dysregulation of STAT3 can have a significant impact on both the worsening and improvement of AD symptoms. In HaCaT cells, the co-stimulation with TNF-α and IFN-γ leads to the activation of both STAT1 and STAT3 [63,64]. Previous studies have identified the inflammatory responses mediated by STAT1 and STAT3 as key targets for kahweol’s anti-inflammatory action in various cells [55,58]. Informed by these studies, we focused on STAT1/3 proteins as kahweol’s main targets and explored its potential to suppress their activities. The findings of our study indicate that in keratinocytes activated with TNF-α/IFN-γ, kahweol effectively decreases the phosphorylation of both STAT1 and STAT3. IF staining corroborated the nuclear translocation of these proteins, and EMSA results emphasized kahweol’s inhibition of the DNA-binding activities of STAT1 and STAT3. Collectively, our results indicate that kahweol effectively hinders the activation of STAT1 and STAT3 in inflamed keratinocytes.

This study has several key limitations that should be addressed in future research. Firstly, we did not include positive controls in our experimental setups, except for the cell viability assays. This omission necessitates caution in interpreting the results, as it is essential to demonstrate that the observed effects are specifically due to the intervention rather than other experimental variables. Therefore, future studies should incorporate appropriate positive controls in all analyses to improve the validity and interpretability of the results. Secondly, the absence of in vivo data limits our ability to fully understand the complex interactions and metabolic processes that occur within an organism. To address this gap, we are planning experiments using mouse models of AD to validate the therapeutic potential of kahweol in a physiological context. Lastly, given the diversity in dietary patterns and genetic makeup across different populations [65,66], the efficacy and safety of natural compounds like kahweol may not be uniform. Future studies are necessary to explore the effects of kahweol in diverse ethnic groups to fully ascertain its clinical relevance and tailor therapeutic strategies accordingly. These steps will ensure a more comprehensive evaluation of kahweol’s potential as a treatment for inflammatory skin conditions.

## 5. Conclusions

In conclusion, our data demonstrate that kahweol effectively reduces inflammation in keratinocytes by suppressing MAPK, NF-κB, and STAT signaling cascades. This study enhances our understanding of kahweol, a primary bioactive component of coffee, and its potential role in dermatological therapies for treating inflammatory skin conditions.

## Figures and Tables

**Table 1:** List of primers.

Gene	Primer Sequence (5′→3′)	Accession No.
*IL-1β*	F: ATGCACCTGTACGATCACTG R: ACAAAGGACATGGAGAACACC	NM_000576
*IL-6*	F: CCACTCACCTCTTCAGAACG R: CATCTTTGGAAGGTTCAGGTTG	NM_000600
*CXCL8*	F: CTGCGCCAACACAGAAATTA R: ATTGCATCTGGCAACCCTAC	NM_000584
*MDC*	F: AGGACAGAGCATGGCTCGCCTACAGA R: TAATGGCAGGGAGGTAGGGCTCCTGA	NM_002990
*GAPDH*	F: GTCTCCTCTGACTTCAACAGCG R: ACCACCCTGTTGCTGTAGCCAA	NM_002046

## Data Availability

The data supporting the findings of this study are available within the article.
